# Diarrhea in an infant due to *Shigella flexneri* 1 carrying multiple cephalosporinase-encoding genes

**DOI:** 10.1186/s13099-021-00413-9

**Published:** 2021-03-20

**Authors:** M. John Albert, Prashant Purohit, Laurent Poirel, Glen Carter, Dieter Bulach

**Affiliations:** 1 Department of Microbiology, Faculty of Medicine, Kuwait University, Jabriya, Kuwait; 2 Department of Medical Microbiology, Al-Sabah Hospital, Shuwaikh, Kuwait; 3 Emerging Antibiotic Resistance Unit and Medical and Molecular Microbiology, Faculty of Science and Medicine, University of Fribourg, Fribourg, Switzerland; 4 Swiss National Reference Center for Emerging Antibiotic Resistance (NARA), University of Fribourg, Fribourg, Switzerland; 5 INSERM European Unit (IAME, France), University of Fribourg, Fribourg, Switzerland; 6 Microbiological Diagnostic Unit, Public Health Laboratory, Peter Doherty Institute for Infection and Immunity, The University of Melbourne, Victoria, Australia

**Keywords:** *S. flexneri* 1, Diarrhea, Multidrug-resistance, Cephalosporinase

## Abstract

**Background:**

Infections caused by multidrug-resistant shigellae resistant to broad-spectrum cephalosporins are becoming more prevalent in the Middle East. We report a case of severe diarrhea due to a multiresistant *Shigella flexneri* 1 strain carrying four different ß-lactamase genes.

**Case presentation:**

A one-year-old Syrian infant presented with severe acute diarrhea, vomiting and dehydration. She did not respond to empirical treatment with amoxicillin-clavulanic acid followed by cefotaxime. Later, stool culture revealed *S. flexneri* 1 resistant to both these drugs. The patient was successfully treated with meropenem to which *S. flexneri* 1 was susceptible. The isolate was resistant to eight classes of antibiotics, and the whole genome sequence (WGS) identified four ß-lactamase genes (*bla*_CTX-M-15_, *bla*_EC-8_, *bla*_OXA-1_, and *bla*_TEM-1_) along with genes mediating resistance to seven other antibiotic classes. The WGS also identified several virulence genes including *senA* that encodes ShET-2 which induces watery diarrhea. Phylogenetically, the isolate was closely related to isolates from South Asia.

**Conclusions:**

This report highlights the emergence of extremely resistant *Shigella* that has acquired multiple resistance genes to cephalosporins rendering these drugs ineffective.

**Supplementary Information:**

The online version contains supplementary material available at 10.1186/s13099-021-00413-9.

## Background

Antibiotics are the cornerstone therapy for treating shigellosis [1]. However, over the years, shigellae have developed resistance to numerous antimicrobials making treatment difficult [2–4]. *Shigella* isolates in many countries have acquired resistance traits leading to the inefficacy of cephalosporins for treating patients [5–9]. Resistance to cephalosporins is mostly mediated by genes located on plasmids and encoding the so-called AmpC or extended-spectrum ß-lactamases (ESBLs) [4, 10, 11]. There are reports of *Shigella* isolates carrying two or three ß-lactamases [8, 12–14]. We describe here a case of shigellosis in an infant in Kuwait due to a multidrug-resistant *Shigella flexneri* 1 that carried four different ß-lactamase genes along with many other antibiotic resistance genes, resulting in a highly drug-resistant strain. We also performed a phylogenetic analysis of the isolate to find out its relationship with the isolates from other parts of the world.

## Case presentation

A one-year old female Syrian infant was admitted at the Al-Sabah hospital, Kuwait, in April 2019, with an acute onset of diarrhea and vomiting, both 8–10 times a day, and fever. There was no macroscopic evidence of blood in the stool. She did not have a history of travel. She was severely dehydrated with a reduced skin turgor and sunken eyes, lethargic and tachypneic, with a body temperature of 40 °C, and a blood pressure of 97/58 mm. She was rehydrated with intravenous fluid containing saline, dextrose and KCl, and treated with intravenous amoxicillin-clavulanic acid (500 mg every 12 h). Her stool, urine and blood samples were analyzed for bacterial culture and susceptibility. Even though her general condition significantly improved after 24 h, fever (39.1 °C) and diarrhea continued. Amoxicillin-clavulanic acid was stopped and intravenous cefotaxime (275 mg every 6 h) was started. Fever (39.7 °C) and diarrhea continued through to the fourth day, when the stool culture and susceptibility report was received (see below). Based on the report, cefotaxime was stopped and intravenous meropenem was started (200 mg every 8 h). After 48 h, the child became afebrile and the frequency of diarrhea was reduced. After the completion of a 7-day course of meropenem, the child was discharged with a complete recovery from diarrhea. Urine and blood cultures were sterile (see below).

Stool, urine and blood samples were cultured for bacterial pathogens as described previously [15]. *S. flexneri* 1 (further designated as SF1) was cultured from stool with no bacteria cultured from other samples. The organism was tested for susceptibility to several antibiotics (HiMedia) by disk diffusion method and interpreted according to CLSI criteria [16]. The isolate was resistant to streptomycin, ampicillin, piperacillin, amoxicillin/clavulanic acid, ampicillin/sulbactam, aztreonam, tetracycline, trimethoprim, sulfamethoxazole, cotrimoxazole, ciprofloxacin, chloramphenicol, cephalothin, cephazolin, cefuroxime, cefotaxime, ceftriaxone, ceftazidime, and cefepime, but susceptible to gentamicin, amikacin, piperacillin/tazobactam, azithromycin, colistin, fosfomycin, ertapenem, meropenem, imipenem, cefoxitin, and tigecycline. These results were corroborated with Etests for all antibiotics except for cephalothin and glycylglycine for which Etests were not available. The isolate was, therefore, multi-resistant to eight classes of antibiotics.

Agar-based double-disk synergy testing showed that the *S. flexneri* 1 isolate produced an extended-spectrum β-lactamase (ESBL) [17].

**Fig. 1:**
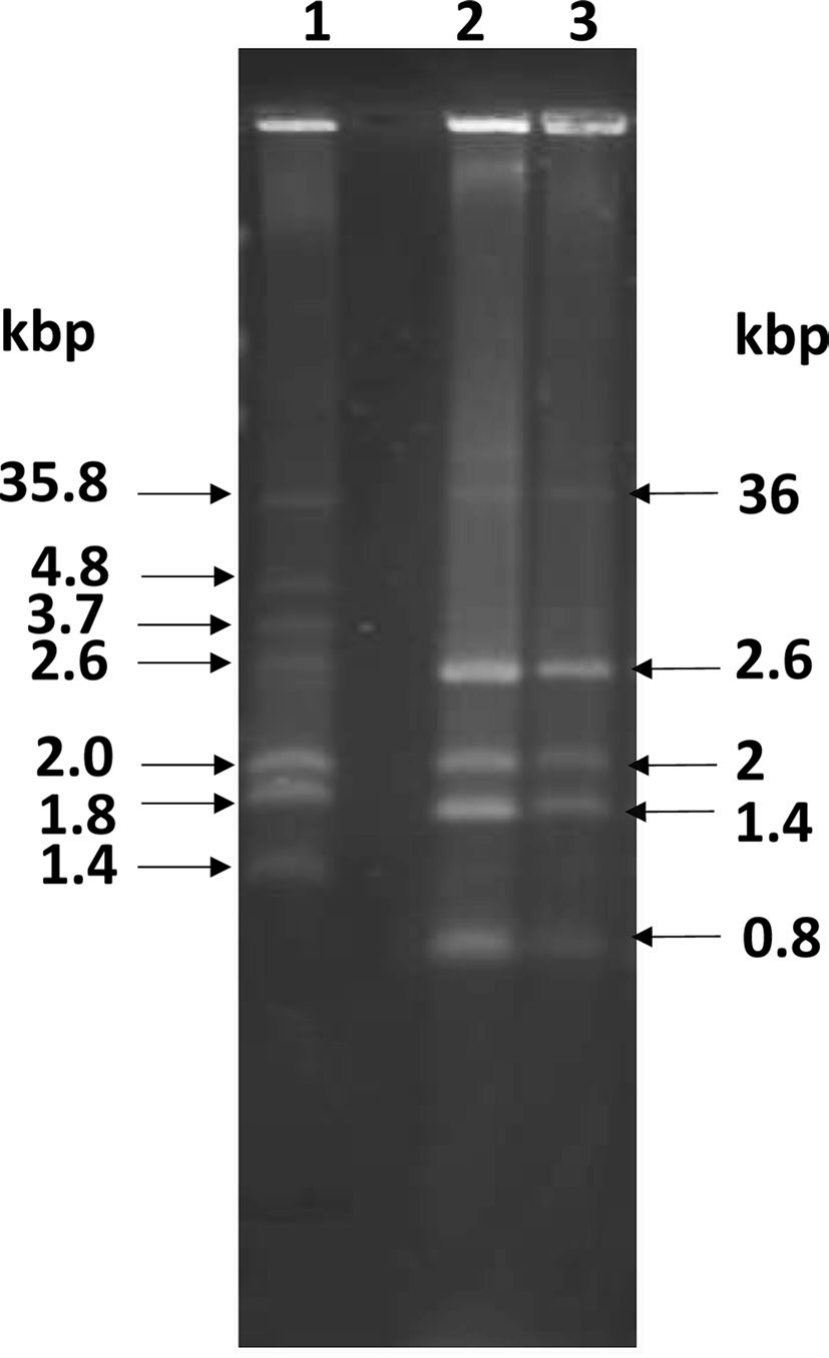
Plasmids present in *S. flexneri* 1. Plasmids were separated by agarose gel electrophoresis, stained with ethidium bromide and photographed under the UV light. Plasmid sizing in *S. flexneri* 1 (lanes 2 & 3) was done in comparison to the known plasmids present in *E. coli* V517 (lane 1)

Chromosomal DNA from *S. flexneri* 1 was extracted by QIAamp Fast DNA Stool Mini Kit (Qiagen). Extraction of plasmids from *S. flexneri* 1 was done with the Plasmid Mini Kit (Qiagen). Plasmids were separated by horizontal gel electrophoresis. Plasmid sizing was done by comparison with plasmids present in *E. coli* V517 (35.8, 4.8, 3.7, 2.6, 2.0, 1.8 and 1.4 kbps). *S. flexneri* 1 had plasmids of sizes 36, 2.6, 2, 1.4 and 0.8 kbp respectively (Fig. 1).

Chromosomal DNA was sequenced. Sequencing libraries were prepared using the Nextera XT DNA sample preparation kit (Illumina) and the sequence read data were produced on the Illumina NextSeq instrument (paired end, 150 base reads). The reads were assembled using Skesa 2.3.0 [18]. The draft assembly contained 418 contigs and 4,412,722 total bases. The sequence type (ST) of the isolate was 245 which confirmed it as *S. flexneri* [19, 20]. Comparative core genome phylogenetic analysis was performed using Nullarbor (https://github.com/tseemann/nullabor). Abricate (https://github.com/tseemann/abricate) was used to detect virulence genes by scanning with VFDB [21] and antibiotic resistance genes by scanning with the Resfinder database [22] and the National Database of Antibiotic Resistant Organisms (NDARO) (https://www.ncbi.nlm.nih.gov/pathogens/antimicrobial-resistance/). Read data produced for the isolate, SF1 has been submitted to NCBI under BioProject, PRJNA556301.

**Fig. 2:**
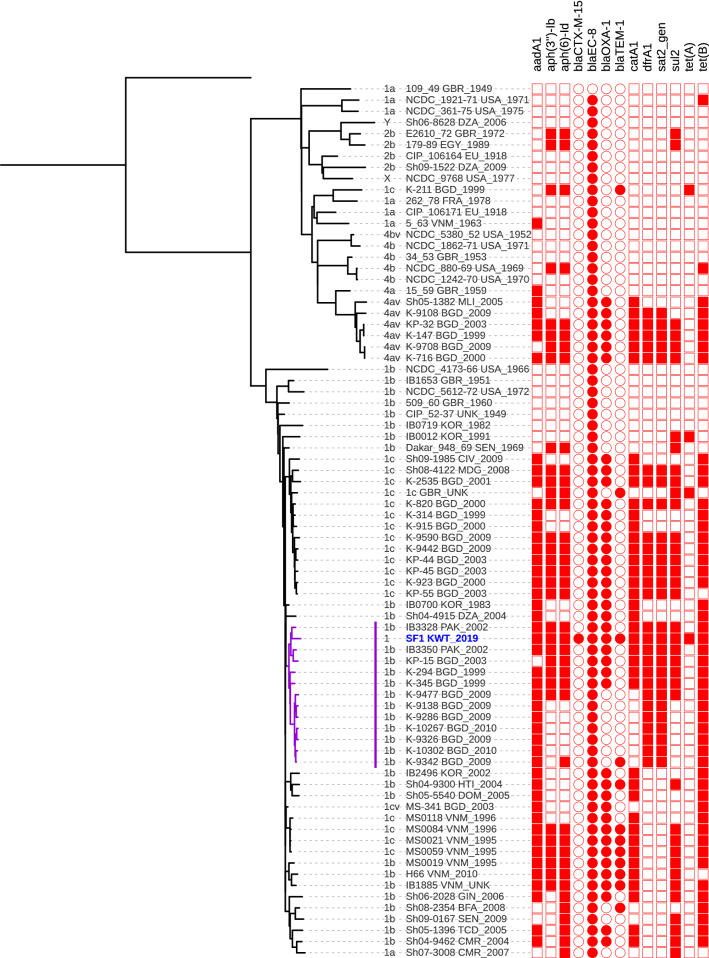
A tree showing the relationship between the core genomes of 77 phylogroup 1 *S. flexneri* isolates from Connor et al. [23] and isolate SF1 (in blue) (metadata can be found in Additional file 1: Table S1). The *S. flexneri* 2a str. 301 genome sequence (Chr: NC_004,337 and pCP301: NC_004851) was used as a reference for the core genome alignment and is represented in the tree as the unlabeled outgroup. The tree was inferred using IQtree version 1.6.12. Approximately, 80% of the *S. flexneri* 2a str. 301 genome sequence was used for the core genome and the greatest pairwise SNP distance was 2151 bases. Taxon labels include serotype, isolate name, country (abbreviated using ISO3166 three letter code; note EU = Europe, and UNK = Unknown) and year of isolation. The close relationship of the Kuwaiti isolate with those from Pakistan and Bangladesh is indicated in purple lines. A genotypic antimicrobial resistance profile for 13 resistance genes is shown at the right of the taxon label where an open box or circle (for β-lactamases) indicates gene absence and closed one indicates gene presence. The figure was produced using the interactive tree of life website (https://itol.embl.de)

The details of phylogroup 1 *Shigella* isolates [23] used for core genome phylogenetic analysis are shown in Additional file 1: Table S1. The phylogenetic tree is shown in Fig. 2. The isolate SF1 clustered with the isolates that had been recovered from Pakistan and Bangladesh. The resistance genes found in the WGS were *aadA1*, *aph(3′’)-1b*, *aph (6)-1d, catA1, dfrA1, qnrS1, sul2, tet(A), tet(B), bla*_CTX-M-15,_ *bla*_EC-8,_ *bla*_OXA-1,_ and *bla*_TEM-1_. No mutation in *gyrA*, *gyrB*, *parC* and *parE* known to confer resistance to quinolone [24] was identified. The resistance genes of the isolates are shown on the right of Fig. 2. The number of resistance genes among the isolates from developing countries appeared to be higher than that in developed countries. A number of virulence genes were identified in SF1, namely *ipaA-ipaD, spa29, ipaH, sepA* and *senA.*

Conjugation experiments for resistance transfer were done by both liquid mating [25] and filter mating [26] with sodium azide-resistant *E. coli* J53 as the recipient strain. Bacterial suspension from the filter and culture after liquid mating were streaked on three MacConkey agar (Oxoid) plates, each one with one of the drug combinations, (sodium azide [200 mg/L] + cefotaxime [2 mg/L], sodium azide [200 mg/L] + ampicillin [50 mg/L], and sodium azide + cefotaxime + ampicillin) to get isolated colonies. No colonies grew on these three selective agars after liquid and filter mating showing a lack of transfer of resistance.

### Discussion and conclusions

Although shigellosis is a typically dysenteric illness with blood, pus and mucus in the stool [27], children often present with diarrhea only [28]. Our patient had watery diarrhea. *Shigella* enterotoxin 1 (ShET-1) and ShET-2 alter water and electrolyte transport in the small intestine, and cause diarrhea and dehydration [29]. Our isolate possessed the *senA* gene that encodes ShET-2 which likely contributed to the severe watery diarrhea. We detected *ipaH* and *spa29* genes that are involved in the invasion of the colonic epithelial cells [30]. The genome also contained the *sepA* gene, which encodes serine protease A autotransporter, which is responsible for barrier disruption [31].

There was a good correlation between the resistance genotypes and phenotypes. The genome contained *aadA1*, *aph(3′’)-1b*, and *aph(6)-1d* all encoding resistance to aminoglycosides, including streptomycin [32, 33]. The isolate also had *catA1, dfrA1, qnrS1*, *sul2*, and *tetA & tetB* genes that mediate resistance to chloramphenicol [34], trimethoprim [35], ciprofloxacin [36], sulfamethoxazole [37], and tetracycline [38] respectively. Phenotypically, the isolate was resistant to all these antibiotics.

The isolate was unusual as it had four different genes encoding β-lactamases belonging to Ambler class A (*bla*_CTX-M-15_, *bla*_TEM-1_), Ambler class C (*bla*_EC-8_) and Ambler class D (*bla*_OXA-1_) [39]. *bla*_CTX-M_ mediates resistance to cefotaxime and ceftriaxone [40, 41], *bla*_EC-8_ mediates resistance to cephalosoprins including third (ceftazidime) and fourth generation (cefepime) cephalosporins [42], and *bla*_TEM-1_ mediates resistance to ampicillin [43]. Accordingly, the isolate possessed phenotypic resistance to these antimicrobials. *bla*_OXA-1_ gene was present in the isolate. This gene mediates resistance to aminopenicillin (ampicillin), ureidopenicillin (piperacillin), and cephalosporin (cefepime) [42]. There is a co-occurrence of *bla*_OXA-1_ with *bla*_CTX-M-15_ in *E. coli* [44]. This co-occurrence makes the isolate resistant to β-lactam–β-lactamase inhibitor combination antibiotics, such as amoxicillin-clavulanic acid and ampicillin-sulbactam [42]. Indeed, this isolate was resistant to these inhibitor combination antibiotics.

The *bla*_CTX-M-15_ gene is present in the chromosome or plasmid in shigellae [45, 46]. The *bla*_EC-8_ gene was reported in *Escherichia coli* with the apparent location of the gene in the chromosome [47]. *bla*_OXA-1_ has been found in plasmid and integron in shigellae [48, 49]. The *bla*_TEM-1_ is present in plasmid or the chromosome [6, 50]. We could not transfer any of the cephalosporinase genes to the recipient *E. coli* by mating, indicating that their locations might be in the chromosome, even though SF1 had plasmids.

Although the child was of Syrian origin, her family had been living in Kuwait long-term with no history of recent travel outside of Kuwait, indicating that the infection was acquired locally. Phylogenetic analysis showed that SF1 was closely related to the isolates from Pakistan and Bangladesh. An expatriate population from these countries resides in Kuwait. It appears that the clone(s) circulating in South Asia is circulating in Kuwait. The study of Connor et al. [23] suggested local acquisition of antimicrobial resistance determinant(s) by isolates in each country, and no evidence of intercontinental spread of antimicrobial resistant strains. However, our study suggested the possible spread of a resistant clone from South Asia to the Middle East. The present day intercontinental migration of refugees portends a widespread transfer of resistant strains.

The occurrence of multiple β-lactamase-encoding genes in *Shigella* is novel which will render cephalosporin antibiotics ineffective for treating the disease.

## Supplementary Information

**Additional file 1: Table S1.** The 77 phylogroup 1 *S. flexneri* 1 isolates used for construction of phylogenetic tree in Fig. 2 are listed. These isolates were taken from Connor et al. [23].

## Data Availability

Sequence data are available in the NCBI BioProject database under the accession number, PRJNA556301. All other data are available in the manuscript.
